# Impaired Chaperone‐Mediated Autophagy Accelerates Intervertebral Disc Degeneration by Inducing MIDN Accumulation to Target TSC2 for Proteasomal Degradation

**DOI:** 10.1002/advs.77428

**Published:** 2026-08-27

**Authors:** Xianglong Chen, Haiyang Gao, Wang Wu, Pengzhi Shi, Yuhang Chen, Anran Zhang, Zhangrong Cheng, Wenbo Wu, Zimu Yu, Yukun Zhang

**Affiliations:** ^1^ Department of Orthopedics Union Hospital Tongji Medical College Huazhong University of Science and Technology Wuhan China

**Keywords:** autophagy, cellular senescence, intervertebral disc degeneration, low back pain, proteostasis

## Abstract

Intervertebral disc degeneration (IDD) is a leading cause of low back pain with incompletely understood mechanisms. Although autophagy dysfunction is a documented contributor to IDD, the precise pathobiological role of chaperone‐mediated autophagy (CMA) remains poorly understood. Here, we demonstrate that CMA activity is downregulated in nucleus pulposus cells (NPCs) from IDD patients and IL‐1β‐induced rat intervertebral disc cell models, causing cytoplasmic accumulation of a novel CMA substrate, Midnolin (MIDN). Accumulated MIDN bypasses the ubiquitin‐proteasome system and directly binds to Tuberous Sclerosis Complex 2 (TSC2), mediating its degradation. TSC2 loss relieves mechanistic target of rapamycin complex 1 (mTORC1) inhibition, resulting in mTORC1 hyperactivation, which drives cellular senescence, senescence‐associated secretory phenotype (SASP), and extracellular matrix (ECM) degradation in NPCs. In vitro and in a rat caudal needle puncture model, MIDN knockdown (shRNA), CMA activation (LAMP2A overexpression), or mTORC1 inhibition (Rapamycin) significantly attenuated IL‐1β or MIDN overexpression‐induced senescence and disc degeneration. Our findings reveal an “Impaired CMA–MIDN accumulation–TSC2 degradation–mTORC1 activation” axis central to IDD pathogenesis, offering potential therapeutic targets.

## Graphical abstract

This graphical abstract illustrates how chaperone‐mediated autophagy (CMA) regulates intervertebral disc degeneration (IDD). Under normal homeostasis (B), CMA degrades cytoplasmic Midnolin (MIDN) to maintain proteostasis. Under inflammatory stress (A), impaired CMA leads to cytoplasmic MIDN accumulation. Accumulated MIDN binds and targets TSC2 for ubiquitin‐independent proteasomal degradation, triggering mTORC1 hyperactivation, SASP secretion, and disc senescence.

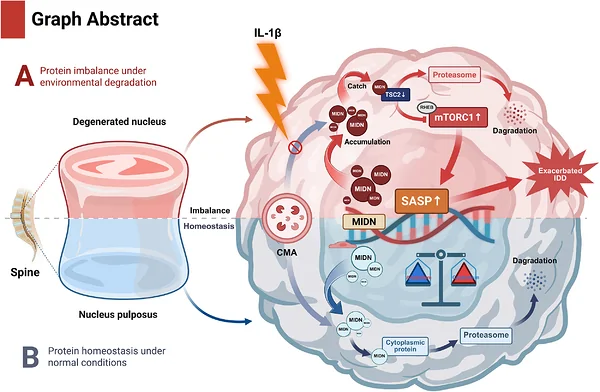

## 1 Introduction

Intervertebral disc degeneration (IDD) is one of the leading causes of chronic low back pain and disability globally, imposing a heavy economic burden on society [1]. Clinically, patients afflicted with IDD manifest debilitating symptoms ranging from persistent, deep discogenic low back pain to severe neurological radiculopathy and motor deficits, which drastically compromise quality of life and workplace productivity. Radiologically, this degenerative cascade is inherently characterized by a progressive narrowing of the intervertebral disc height on plain radiographs, coupled with a profound loss of T2‐weighted signal intensity (the “black disc” phenomenon) and structural collapse on magnetic resonance imaging (MRI), fundamentally reflecting the matrix dehydration and collapse of the NP space. The pathological features of IDD mainly include a reduction in NP cells, an imbalance between the degradation and synthesis of the ECM, and increased cell senescence and apoptosis. Although various factors, such as aging, genetics, mechanical stress, inflammation, and oxidative stress, have been confirmed to participate in the occurrence and development of IDD, its core molecular mechanisms have not yet been fully elucidated [2]. Consequently, current clinical treatments primarily focus on transient symptom relief via conservative standard‐of‐care regimens (such as non‐steroidal anti‐inflammatory drugs and physical therapy) or invasive surgical modalities (such as discectomy and spinal fusion). However, these approaches remain palliative, fail to restore the biological functionality of the degenerated disc, and frequently accelerate adjacent segment degeneration, highlighting a critical diagnostic and therapeutic void that lacks effective interventions targeting the etiology.

Maintaining proteostasis is crucial during the complex pathological process of IDD. As a relatively “closed” system, the balance of protein synthesis and degradation in NP cells is vital for maintaining cell function and ECM integrity [3]. The autophagy‐lysosome pathway and the ubiquitin‐proteasome system (UPS) are the two major intracellular protein degradation pathways [4, 5]. Accumulating evidence indicates that a decline in autophagic activity is closely related to the severity of IDD. Impaired autophagy leads to the accumulation of dysfunctional organelles and abnormal proteins, inducing cell senescence and the SASP phenotype, thereby accelerating the IDD process [6]. Recent authoritative reviews within spinal research have progressively converged on the concept that general autophagy decline serves as an upstream master switch driving nucleus pulposus cell senescence and the transition to a catabolic secretory state [7, 8]. This consensus underscores that chronological aging and inflammatory stress collaboratively disrupt macroautophagic flux, converting cellular defense mechanisms into chronic senescence‐associated cascades within the degenerating spinal microenvironment [9, 10, 11]. While these advances highlight macroautophagy as a pivotal checkpoint, the selective contributions of specialized lysosomal pathways under these senescent trajectories remain an evolving paradigm.

CMA is a highly specific form of autophagy that recognizes substrate proteins containing a KFERQ motif via molecular chaperones (such as HSC70) and transports them to lysosomes (via the LAMP2A channel) for degradation [12]. CMA plays a refined regulatory role in responding to stress, maintaining proteostasis, and regulating cell fate [13, 14]. Our previous studies have confirmed that CMA function is impaired in IDD and mediates the accumulation of specific substrates (such as PLGC1) [15]. However, the complete substrate profile of CMA dysfunction in IDD and its downstream pathogenic mechanisms remain areas urgently needing exploration.

MIDN is a recently highlighted protein reported to contain a ubiquitin‐like (Ubl) domain and two Catch domains, possibly participating in mediating non‐ubiquitin‐dependent proteasomal degradation pathways [16]. As a master orchestrator of cell fate, the downstream activity of the proteasome system is intricately linked to TSC2—a canonical upstream negative regulator and indispensable molecular brake of the mTORC1 signaling cascade [17, 18]. Under biological homeostasis, the intact TSC2 complex restrains hyperactivation of downstream mTORC1, thereby safeguarding cells against premature senescence and matrix catabolism [18]. Although the individual roles of TSC2/mTORC1 signaling and MIDN‐dependent proteasomal clearance have been distinctively characterized in disparate pathological contexts, whether a direct molecular biochemical intersection exists between MIDN accumulation and TSC2 stability in the pathogenesis of metabolic bone disorders remains completely uncharacterized.

In this study, through analysis of clinical IDD samples and in vitro inflammatory models, we discovered for the first time that MIDN is not transcriptionally upregulated in IDD but rather accumulates abnormally due to impaired CMA function. We further revealed that accumulated MIDN activates the mTORC1 signaling pathway by mediating the degradation of TSC2 [19], ultimately driving NP cell senescence and intervertebral disc degeneration. This study not only elucidates the novel CMA–MIDN–TSC2–mTORC1 signaling axis but also provides a new theoretical basis and potential therapeutic targets for the prevention and treatment of IDD.

## 2 Result

### 2.1 MIDN Induces Degenerative Phenotypes in Nucleus Pulposus Cells at Both Transcriptional and Protein Levels

Intracellular proteostasis imbalance is a key factor in the development of IDD. To investigate the specific role of the novel protein degradation pathway mediator MIDN in NPCs, we constructed MIDN overexpression (OE‐MIDN) lentiviruses and transfected them into normal rat NPCs. Systematic analysis was subsequently performed via transcriptomics (RNA‐Seq) and proteomics (Figure 1a). The transcriptomic volcano plot (Figure 1b) showed that MIDN overexpression caused drastic remodeling of the gene profile. Gene Set Enrichment Analysis (GSEA) further revealed that differential genes were significantly enriched in pathways highly relevant to IDD pathology, including “Response to Interleukin‐1,” “Cell Senescence,” and “Extracellular Matrix Structural Organization” (Figure 1c). KEGG pathway clustering heatmaps (Figure 1d,e) also showed significant activation of the “p53 signaling pathway,” “TGF‐β signaling pathway,” and “mTOR signaling pathway.” The proteomics volcano plot (Figure 1f) and GO functional enrichment analysis (Figure 1g,h) similarly confirmed that differentially expressed proteins were mainly concentrated in functions such as “Extracellular matrix organization” and “Collagen‐containing extracellular matrix.” To identify the core regulatory hub, a Protein‐Protein Interaction (PPI) network was constructed (Figure 1i), which clearly pointed to the mTORC1 signaling pathway centered on “TSC2‐RHEB‐MTOR.” This pathway is not only closely interconnected with key inflammatory/SASP factors (e.g., TNF, IL6, IL1B) but also linked to various matrix‐degrading metalloproteinases (MMP3, MMP9, MMP13) [20]. By integrating transcriptomic and proteomic data, differential genes and proteins were found to be highly enriched in pathways related to cell matrix synthesis, such as “Extracellular matrix degradation” (Figure 1j).

**FIGURE 1:**
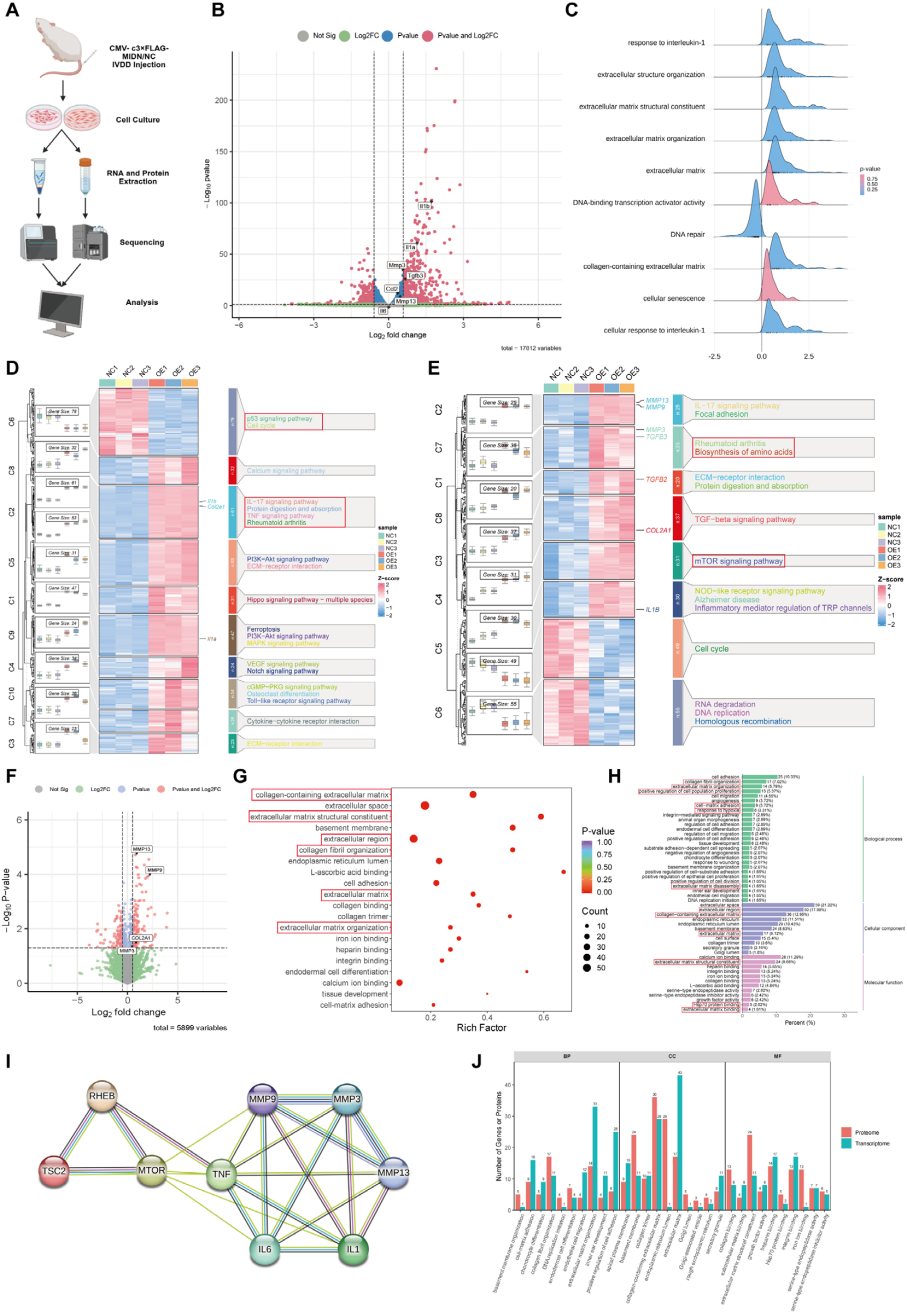
MIDN Induces Degenerative Phenotypes in Nucleus Pulposus Cells at Both Transcriptional and Protein Levels. (a) Schematic diagram of the experimental workflow: Construction of OE‐MIDN lentivirus for transfection into rat NPCs, followed by RNA‐Seq and proteomics analysis. (b) RNA‐Seq volcano plot showing differentially expressed genes (DEGs) between the OE‐MIDN group and the control group (CT). (c) Ridge plot of Gene Set Enrichment Analysis (GSEA), showing pathways significantly enriched in the OE‐MIDN group, such as response to IL‐1, cell senescence, and ECM structural organization. (d, e) Heatmaps of KEGG pathway enrichment for DEGs (d) and DEPs (e), indicating activation of signaling pathways including mTOR, p53, and TGF‐β. (f) Proteomics volcano plot showing differentially expressed proteins (DEPs) between the OE‐MIDN and CT groups. (g, h) GO functional enrichment analysis (bubble plot and bar chart) of DEPs, showing that differential proteins are mainly concentrated in ECM‐related functions. (i) PPI network of DEPs, highlighting the regulatory hub centered on mTORC1 (MTOR, RHEB, TSC2). (j) Schematic of pathway enrichment integrating transcriptomic and proteomic differential data. Data represent *n* = 3 biological replicates.

These findings indicate that MIDN is not merely a bystander of cellular degeneration, but rather a functional driver of IDD pathogenesis. Simply elevating MIDN levels is sufficient to recapitulate core IDD pathological features (senescence and matrix degradation) in healthy cells, and unbiased PPI network analysis directly points its pathogenic mechanism to the mTORC1 signaling axis. This suggests that MIDN may act as a critical node connecting upstream stress signals to downstream mTORC1 metabolic reprogramming, “hijacking” the cell's metabolic regulatory center through a specific molecular interaction mechanism.

### 2.2 MIDN is a Critical Molecule Aggravating IDD Progression

Based on the omics analysis hints, we first examined MIDN expression in clinical IDD nucleus pulposus specimens. To obtain non‐degenerated controls, scoliosis patients undergoing anterior surgery provided Pfirrmann Grade I‐II specimens, while degenerated disc tissues (Pfirrmann Grade IV‐V) were collected from lumbar disc omission patients. Western blot (WB) experiments were performed on nucleus pulposus cells extracted from specimens with varying degrees of degeneration. Results showed that MIDN protein levels were significantly positively correlated with IDD severity (Pfirrmann grade) (Figure 2a,b). Next, we used IL‐1β to mimic the disc inflammatory degeneration environment to construct a cell model. Through screening of IL‐1β concentration gradients, we determined 20 ng/mL as the optimal concentration. RT‐qPCR (Figure S1a), Western Blot, and Immunofluorescence (IF) results consistently showed that IL‐1β treatment led to increases in protein levels of MIDN (Figure 2c–f), while MIDN mRNA levels decreased (Figure 2e). This critical “transcript‐protein level inversion” phenomenon suggests that MIDN may undergo blocked post‐translational degradation. Furthermore, the researchers measured intracellular CCK‐8 levels over a period of 24 to 72 h, and the results demonstrated that treatment with 20 ng/mL IL‐1β did not induce acute cytotoxicity in rat NPCs, and the overexpression of MIDN did not further compromise cell viability compared to the IL‐1β stimulation alone (Figure 2g).

**FIGURE 2:**
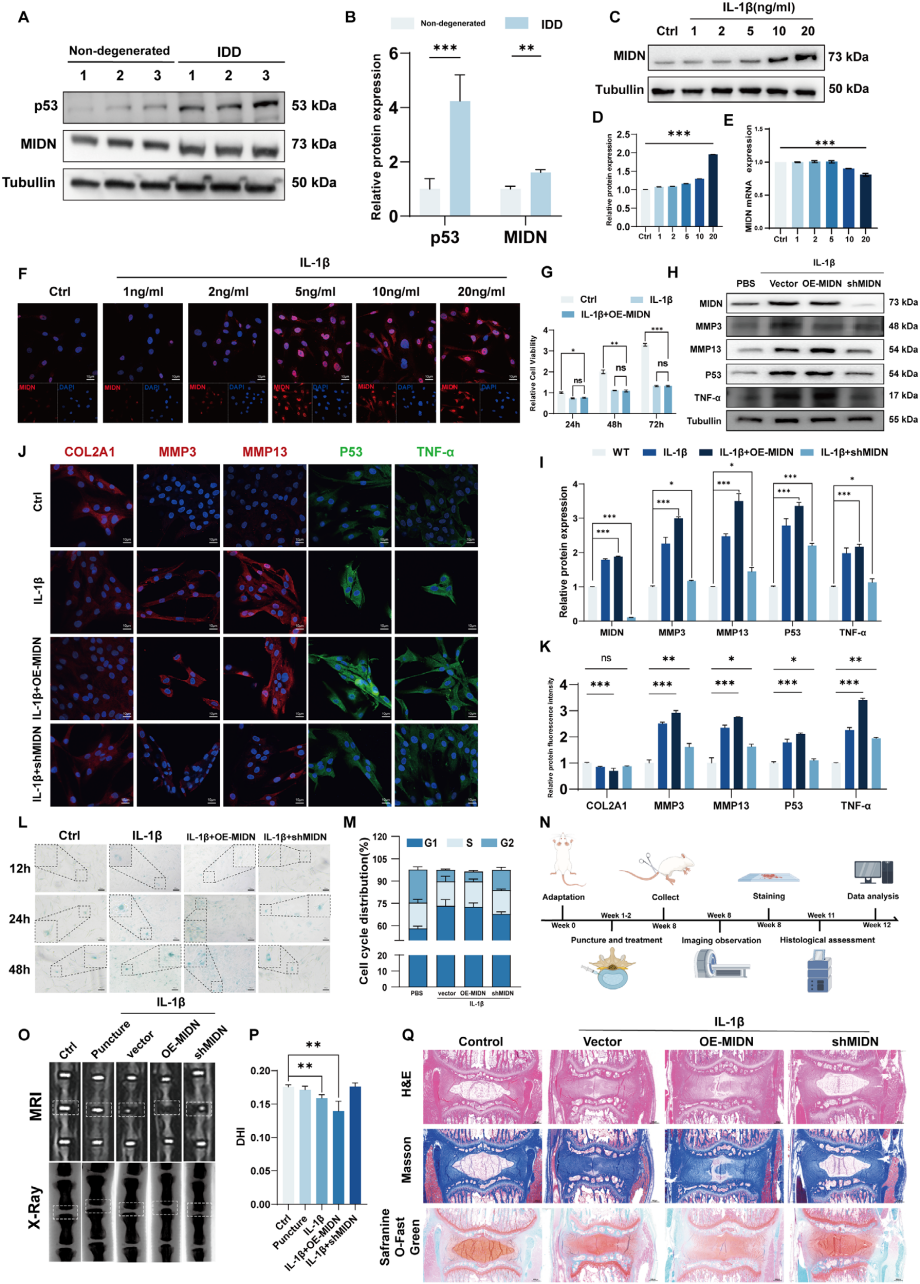
MIDN is a Critical Molecule Aggravating IDD Progression. (a, b) Western blot (WB) detection and quantification of MIDN and cellular senescence marker p53 in human NP tissues with different Pfirrmann grades (I–II for non‐degenerated controls, and IV–V for IDD). (c, d) WB detection and quantification of p53 and MIDN in rat NPCs treated with IL‐1β (0, 1, 2, 5, 10, 20 ng/mL). (e) RT‐qPCR detection of MIDN mRNA levels in human IDD specimens and IL‐1β‐treated NPCs. (f) Immunofluorescence (IF) staining and quantification of MIDN in NPCs treated with IL‐1β. (g) CCK‐8 cell viability and proliferation assay of NPCs under Control, IL‐1β, or IL‐1β + OE‐MIDN transfection over 24, 48, and 72 h. (h, i) WB detection and quantification of ECM (COL2A1, MMP13) and senescence marker p53 in NPCs transfected with Vector, OE‐MIDN, or shMIDN under IL‐1β treatment. (j, k) IF staining and quantification of COL2A1, MMP3, MMP13, p53, and TNF‐α in NPCs. (l) SA‐β‐gal staining of NPCs in each group. (m) Flow cytometry cell cycle distribution analysis of NPCs. (n) Schematic representation of the experimental workflow for the rat caudal needle puncture (IDD) model injected with Vector, OE‐MIDN, or shMIDN lentiviruses. (o) x‐ray and T2‐weighted MRI images of rat tails at 8 weeks post‐operation. (p) Quantitative analysis of Disc Height Index (DHI%) at 8 weeks post‐operation. (q) H&E, Masson's trichrome, and Safranin O‐Fast Green (SOFG) staining. Data represent Mean ± SD, *n* = 3. ^*^ *p* < 0.05, ^**^ *p* < 0.01, ^***^ *p* < 0.001. Scale bars are labeled.

To investigate the precise role of MIDN in IDD, we constructed OE‐MIDN (overexpression) and shMIDN (knockdown) plasmids. Under IL‐1β conditions, OE‐MIDN significantly exacerbated matrix degradation (increased MMP3, MMP13; decreased COL2A1) and senescence (increased p53, TNF‐α) (Figure 2h–k and Figure S1a). Conversely, MIDN knockdown significantly reversed these degenerative phenotypes (Figure 2h–k and Figure S1a). SA‐β‐gal staining (Figure 2l and Figure S1b) confirmed that OE‐MIDN aggravated IL‐1β‐induced cell senescence, while shMIDN alleviated this state. Notably, flow cytometry analysis revealed that while MIDN overexpression (OE‐MIDN) alone caused a modest shift in cellular distribution, its combination with IL‐1β (IL‐1β + OE‐MIDN) did not induce a further statistically significant increase in G0/G1 phase arrest compared to the IL‐1β solitary group (Figure 2m and Figure S1c). This absence of an additive phenotype suggests a biological saturation effect, wherein the severe inflammatory stress triggered by 20 ng/mL IL‐1β already drives the cell‐cycle arrest near its physiological maximum, rendering the cell cycle largely refractory to additional exogenous MIDN input. Finally, in the in vivo rat caudal needle puncture model (Figure 2n), MRI and x‐Ray (Figure 2o,p) as well as Pfirrmann grades (Figure S1d) and histological staining (Figure 2q and Figure S1e) indicated that intervertebral disc degeneration was significantly exacerbated by injection of MIDN overexpression lentivirus, whereas shMIDN demonstrated a clear protective effect. The “transcript‐protein level inversion” consistently observed in clinical samples and cell models strongly implies that MIDN accumulation stems from blocked protein degradation pathways rather than increased synthesis. This abnormal post‐transcriptional accumulation constitutes an important pathological basis for IDD pathogenesis. The protective effect of shMIDN further confirms that by cutting off the mRNA source to reduce continuous protein input, the intracellular MIDN load caused by degradation obstacles can be effectively reduced, thereby blocking its downstream pathogenic effects.

### 2.3 MIDN is a Substrate of CMA, and Its Abnormal Accumulation is Caused by Impaired CMA Function

To clarify the upstream molecular mechanism of MIDN protein accumulation, we first examined its subcellular localization under degenerative conditions. Immunofluorescence staining showed that under IL‐1β stimulation, significant pan‐accumulation of MIDN occurred in NPCs, with a particularly marked increase in cytoplasmic signal (Figure 3a,b). Nuclear‐cytoplasmic fractionation Western Blot and its quantitative analysis further confirmed that IL‐1β treatment resulted in a significant increase in MIDN in both nuclear and cytoplasmic fractions (Figure 3c,d).

**FIGURE 3:**
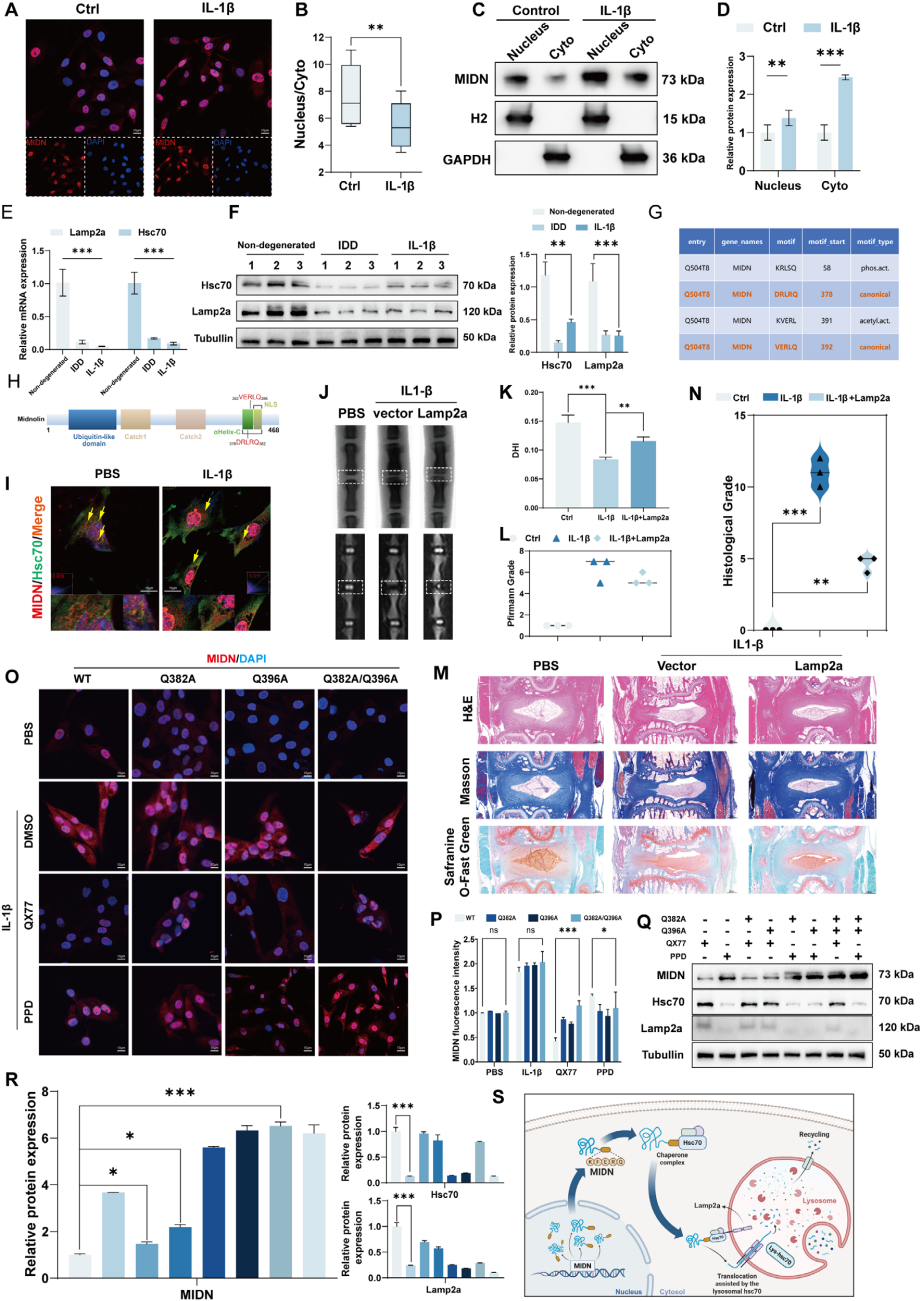
MIDN is a Substrate of CMA, and Its Abnormal Accumulation is Caused by Impaired CMA Function. (a, b) IF staining of MIDN in rat NPCs under IL‐1β treatment, showing significant accumulation in both the cytoplasm and nucleus. (c, d) Nuclear‐cytoplasmic fractionation WB detection (c) and relative expression quantification (d), confirming that IL‐1β treatment leads to increased MIDN in both nuclear and cytoplasmic fractions. (e) RT‐qPCR analysis of Hsc70 and LAMP2A mRNA expression levels in primary rat nucleus pulposus cells under non‐degenerated (Ctrl), IDD, and IL‐1β‐stimulated conditions. (f) WB detection and relative protein quantification of Hsc70 and LAMP2A. (g) Screenshot of KEFRQ finder bioinformatic analysis showing high‐confidence HSC70 binding potential in the MIDN sequence. (h) Schematic structure of MIDN protein highlighting two KFERQ‐like motifs: 378‐DRLRQ‐382 and 392‐VERLQ‐396. (i) IF colocalization of MIDN and HSC70. (j, k, l) In vivo rescue experiments: x‐ray and MRI images (j), Disc Height Index (DHI%) quantification (k), and Pfirrmann grading (l) after injection of PBS, Vector, or OE‐Lamp2a lentivirus in the rat caudal puncture model. (m, n) Histological staining (H&E, Masson, Safranin O‐Fast Green) (m) and histological scoring (n) of discs at 8 weeks post‐operation, showing that activating CMA (Lamp2a) significantly alleviates degeneration. (o, p) IF staining of wild‐type (WT) and KFERQ mutant (Q382A, Q396A, Double Mutant) MIDN, showing more severe accumulation of mutants under IL‐1β stimulation. (q, r) WB detection (q) and quantification (r) show that WT MIDN stability is regulated by CMA activator (QX77) or inhibitor (PPD), while mutant MIDN is insensitive to these treatments. (s) Schematic mechanism: HSC70 recognizes KFERQ motifs on MIDN and assists its translocation into the lysosome via LAMP2A for degradation. Data represent Mean ± SD, *n* = 3. Scale bars are clearly displayed in high resolution.

Subsequently, we investigated whether MIDN accumulation originated from CMA pathway abnormalities. RT‐qPCR and Western Blot results showed that the expression of CMA key rate‐limiting proteins, Hsc70 and LAMP2A, was significantly downregulated in IDD patient tissues and IL‐1β‐treated NPCs at both transcriptional and translational levels (Figure 3e,f), suggesting impaired CMA function [21]. Given that CMA is responsible for clearing cytoplasmic substrates, we hypothesized that MIDN might be a CMA substrate. Analysis using the bioinformatic tool KEFRQ finder suggested it has high‐confidence HSC70 recognition potential (Figure 3g). Further motif mapping identified two classic KFERQ‐like motifs in the MIDN protein sequence: DRLRQ at positions 378–382 and VERLQ at positions 392–396 (Figure 3h). Meanwhile, we demonstrated the interaction in subcellular localization through immunofluorescence colocalization of MIDN and HSC70 (Figure 3i).

Based on the above findings, we deduced the degradation mode of MIDN: cytoplasmic MIDN is specifically recognized by HSC70 via its KFERQ motifs, then transported to the LAMP2A receptor on the lysosomal membrane, and finally translocated into the lysosomal lumen for degradation. In IDD, downregulation of CMA components directly arrests this process, leading to MIDN accumulation.

To verify this mechanism at the functional level, we performed in vivo rescue experiments. Injection of a LAMP2A overexpression lentivirus into rat caudal vertebrae to activate CMA showed that CMA activation significantly reversed IL‐1β‐induced disc degeneration, manifested as recovery of Disc Height Index (DHI) on x‐ray and MRI (Figure 3j,k), improvement in Pfirrmann scores (Figure 3l), and significant improvement in histological structure (H&E, Masson, Safranin O) (Figure 3m,n). We observed that although LAMP2A overexpression significantly restored disc structure, the therapeutic efficacy exhibited some mechanistic limitations under persistent inflammatory stress, and some post‐injury fibrocartilaginous remodeling of the NP was noted in the LAMP2A‐treated group (Figure 3m,n). Molecular specificity verification showed that mutating KFERQ motifs (Q382A, Q396A) allowed MIDN to escape CMA‐mediated degradation, resulting in much higher accumulation under IL‐1β treatment compared to wild type (Figure 3o), and loss of sensitivity to CMA activator (QX77) or inhibitor (PPD) (Figure 3p–r). A schematic diagram of this process is shown in Figure 3s.

### 2.4 MIDN Interacts With TSC2 to Promote Non‐Ubiquitinated TSC2 Degradation

To explore the reason why MIDN accumulation leads to cell senescence, IP‐MS analysis was performed to screen for MIDN‐interacting proteins. The results showed that TSC2 (a key negative regulator of the mTORC1 pathway) is a potential interacting protein of MIDN (Figure 4a,b). Co‐IP and IF colocalization experiments confirmed a strong interaction between endogenous MIDN and TSC2 (Figure 4c,d and Figure S2a). Domain mapping further revealed that the CATCH domain of MIDN is the key region for its binding to TSC2 (Figure 4e,f).

**FIGURE 4:**
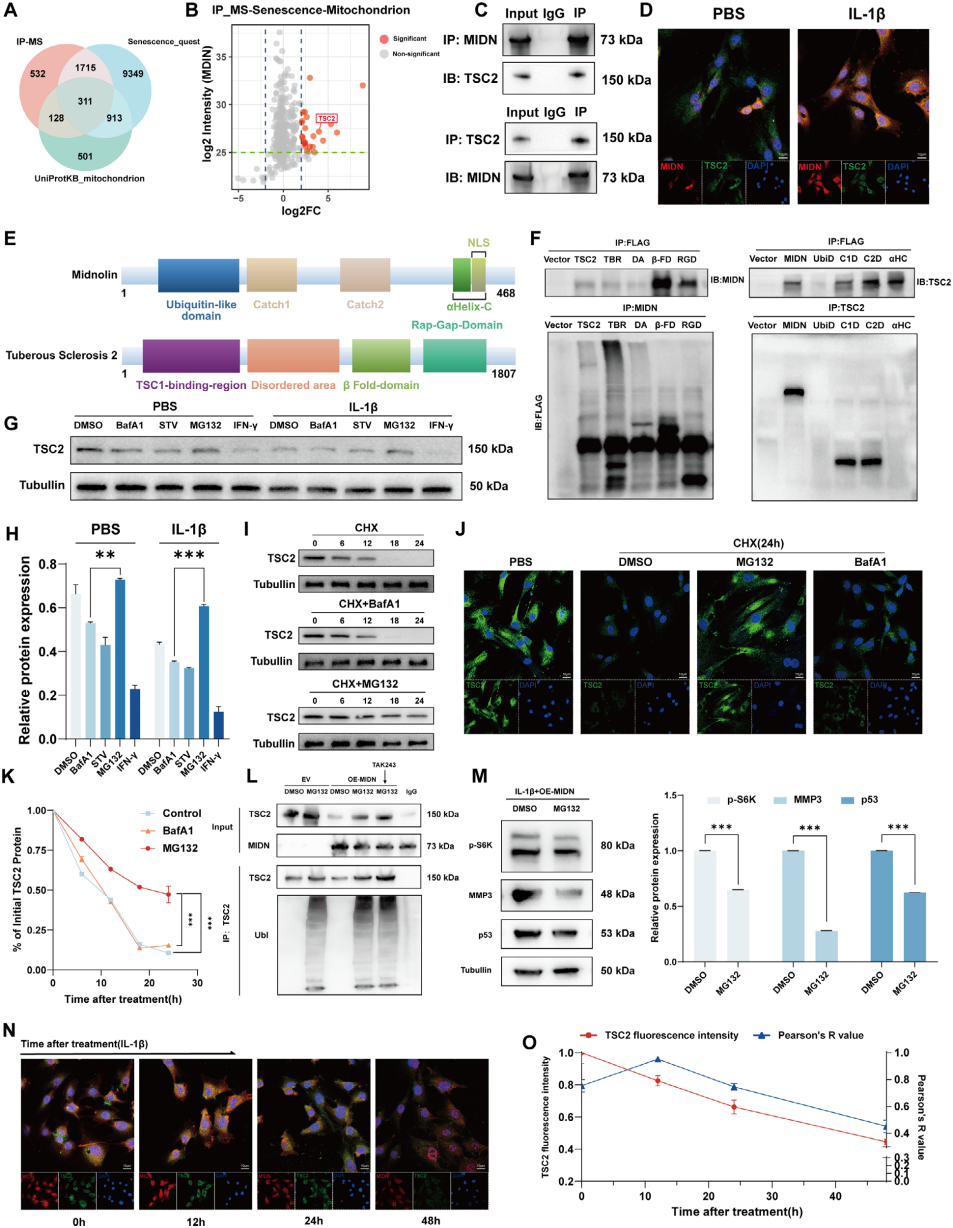
MIDN Interacts with TSC2 to Promote Non‐Ubiquitinated TSC2 Degradation. (a, b) Venn diagram (a) and volcano plot (b) from IP‐MS analysis identifying TSC2 as a potential interacting protein of MIDN. (c) Co‐IP experiment confirming the interaction between endogenous MIDN and TSC2. (d) IF colocalization analysis showing spatial overlap of MIDN and TSC2 in cells. (e) Schematic of MIDN and TSC2 protein domains. (f) Co‐IP domain mapping confirming MIDN binds to TSC2 via the Catch domain. (g, h) WB detection (g) and quantification (h) confirming TSC2 degradation is mainly dependent on the proteasomal pathway (MG132‐sensitive) rather than the lysosome pathway. (i) WB showing TSC2 degradation over time in the presence of CHX, co‐cultured with proteasome or lysosome pathway activators/inhibitors. (j, k) IF detection of the effects of lysosome inhibitor (BafA1) and proteasome inhibitor (MG132) on TSC2 degradation. (l) Endogenous TSC2 ubiquitination assay under proteasome inhibitor MG132 conditions, showing that MIDN overexpression accelerates TSC2 clearance without altering its poly‐ubiquitination status. (m) Western blot and relative expression quantification of downstream target p‐S6K, matrix metalloproteinase MMP3, and cellular senescence marker p53, showing therapeutic rescue by co‐administering low‐toxicity proteasome inhibitor MG132 in the IL‐1β+OE‐MIDN model. (n) IF staining showing spatial localization and intensity of MIDN and TSC2 at different time points of IL‐1β stimulation (0 h, 12 h, 24 h, 48 h). (o) Quantitative analysis of TSC2 fluorescence intensity and Pearson's correlation coefficient (R value) showing the temporal dynamics of MIDN‐TSC2 colocalization. The Pearson correlation coefficient (R‐value) was employed to quantify the spatial co‐localization relationship between MIDN and TSC2. A higher R‐value closer to 1 indicates a stronger degree of actual physical co‐localization, with values > 0.5 considered statistically significant relative to a random background baseline. Data represent Mean ± SD, *n* = 3. ^*^ *p* < 0.05, ^**^ *p* < 0.01, ^***^ *p* < 0.001.

Based on this, a hypothesis was proposed: MIDN accumulation affects TSC2 stability. Interestingly, the proteasome inhibitor MG132 blocked IL‐1β‐induced TSC2 degradation, while the lysosomal inhibitor BafA1 was ineffective (Figure 4g,h). CHX chase assays found that inhibiting the proteasomal pathway significantly slowed the degradation rate of TSC2 protein (Figure 4i), which was also confirmed by TSC2 immunofluorescence after 24 h treatment (Figure 4j,k). Furthermore, we conducted endogenous TSC2 ubiquitination assays under MG132‐mediated proteasome inhibition conditions. Consistent with our hypothesis, the results demonstrated that although MIDN overexpression significantly accelerated TSC2 degradation, the polyubiquitination levels of residual TSC2 remained unchanged compared to the control group. This compelling evidence unequivocally demonstrates that the MIDN‐mediated TSC2 clearance process bypasses the classical ubiquitination mechanism(Figure 4l). Furthermore, we co‐administered low‐toxicity concentrations of MG132 in the IL‐1β+OE‐MIDN cell model and monitored downstream effector proteins by Western blotting. The results unequivocally demonstrated that MG132's proteasome‐inhibitory effect successfully blocked MIDN‐mediated degradation of TSC2, thereby significantly reversing downstream hyperactivation markers (reduced p‐S6K levels) and markedly reducing the expression of catabolic/age‐related markers (MMP3 and p53) (Figure 4m). This robust evidence for therapeutic rescue further elucidates the mechanistic link between TSC2 and its downstream effects mediated by the proteasome. Notably, IF showed that with prolonged IL‐1β treatment time, TSC2 fluorescence intensity showed a downward trend (Figure 4n), while its colocalization with MIDN showed a trend of first rising and then falling (Figure 4o). This aligns with the MIDN nuclear‐cytoplasmic crosstalk hypothesis, where TSC2 interacts with shuttled MIDN intracellularly and is gradually degraded during this interaction.

It is generally believed that TSC2 turnover is regulated by the ubiquitin‐proteasome system (UPS) [22], but our data reveal a non‐canonical degradation mechanism under stress conditions: accumulated MIDN acts as a molecular “shuttle,” binding TSC2 and directing it directly to the proteasome for degradation. This bypass mechanism circumvents conventional phosphorylation regulation, explaining why cells lose control over mTORC1 due to “physical depletion” of TSC2 in an inflammatory environment, even without typical growth factor stimulation. The schematic diagram of the specific mechanism is shown in Figure S2b.

### 2.5 MIDN Activates mTORC1 via TSC2 Degradation

Given that TSC2 is a core negative regulator of the mTORC1 signaling pathway, we inferred that MIDN‐mediated TSC2 degradation would lead to activation of the mTORC1 signaling pathway. Western Blot detection showed that IL‐1β stimulation caused a dose‐dependent activation of the mTORC1 pathway (manifested as significant upregulation of p‐S6K, p‐S6, and p‐4E‐BP1 phosphorylation levels) while decreasing TSC2 protein levels (Figure 5a,b).

**FIGURE 5:**
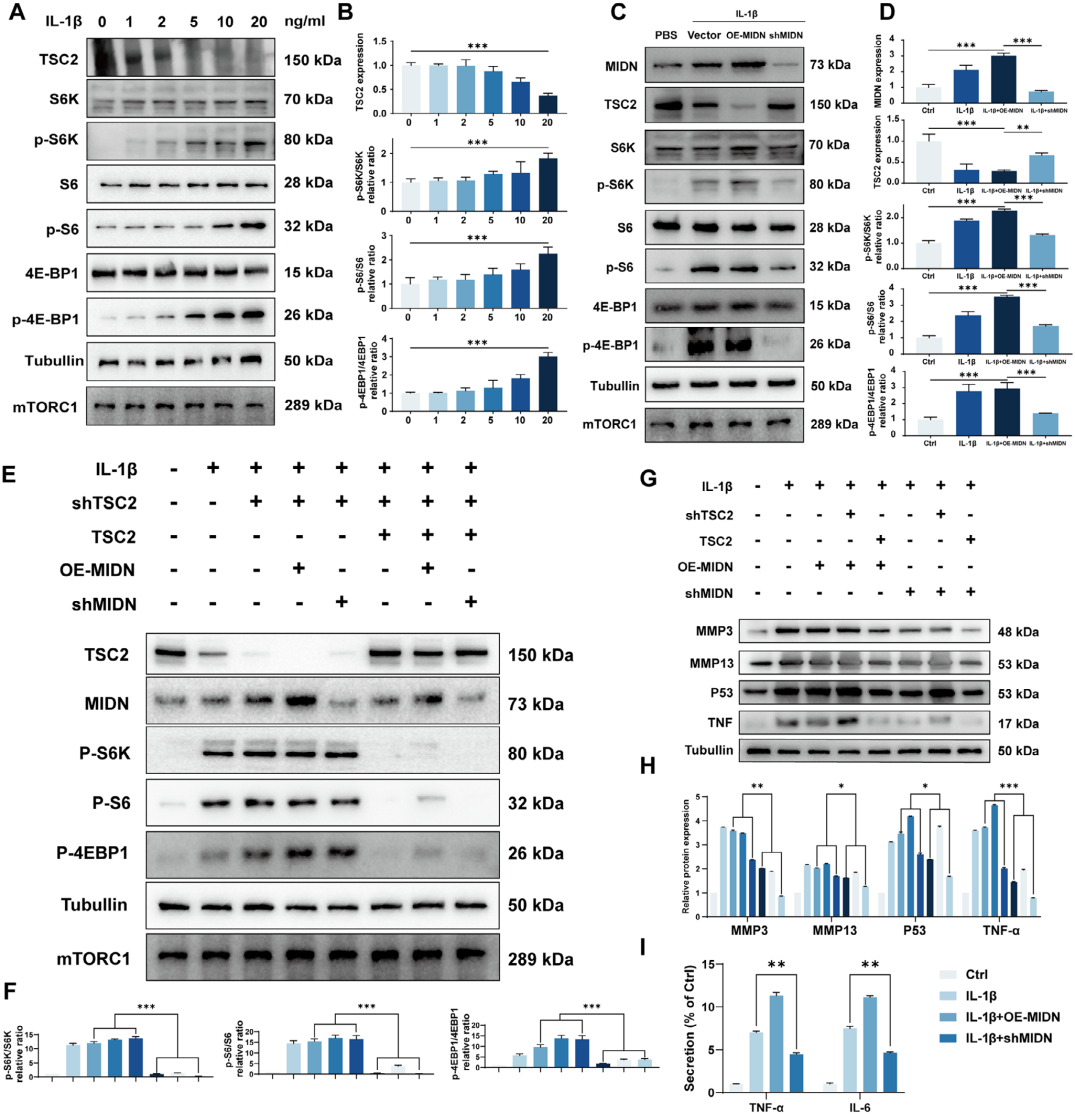
MIDN Activates mTORC1 via TSC2 Degradation. (a, b) WB detection (a) and quantification (b) showing that IL‐1β dose‐dependently decreases TSC2 levels and activates the mTORC1 signaling pathway (p‐S6K, p‐S6, p‐4E‐BP1). (c, d) WB detection (c) and quantification (d) of mTORC1 pathway‐related proteins in NPCs transfected with Vector, OE‐MIDN, or shMIDN under an IL‐1β background. (e, f) WB images (e) and quantification (f) of mTORC1 pathway activity in OE‐MIDN cells co‐transfected with shTSC2 or OE‐TSC2. (g, h) WB images (g) and quantification (h) of ECM markers (MMP3, MMP13) and senescence markers (p53, TNF‐α), confirming that TSC2 restoration reverses degenerative phenotypes caused by OE‐MIDN. (i) ELISA quantitative analysis of extracellular TNF‐α and IL‐6 secretion levels in cell culture supernatants of primary rat NPCs under Ctrl, IL‐1β (20 ng/mL), IL‐1β+OE‐MIDN, or IL‐1β+shMIDN transfection conditions, demonstrating the regulation of SASP paracrine activity by the CMA‐MIDN‐TSC2 axis. Data represent Mean ± SD, *n* = 3. ^*^ *p* < 0.05, ^**^ *p* < 0.01, ^***^ *p* < 0.001.

To clarify whether this activation is MIDN‐dependent, gain‐ and loss‐of‐function experiments were implemented. Under an IL‐1β background, OE‐MIDN further exacerbated TSC2 degradation and significantly enhanced mTORC1 activity (Figure 5c,d). Conversely, shMIDN reversed the IL‐1β‐induced TSC2 decline and inhibited mTORC1 pathway activation (Figure 5c,d).

To verify that TSC2 is the core mediator of MIDN‐induced mTORC1 activation, rescue experiments were performed. Co‐knockdown of TSC2 (shTSC2) in OE‐MIDN cells further amplified mTORC1 activity (Figure 5e,f). However, restoring TSC2 (TSC2 overexpression) in OE‐MIDN cells completely blocked mTORC1 pathway activation induced by OE‐MIDN (Figure 5e,f) and significantly reversed the senescence and matrix degradation phenotypes [23] caused by OE‐MIDN (Figure 5g,h). To obtain direct, physiologically relevant secretion data, we collected culture supernatants from the Ctrl group, IL‐1β group, IL‐1β+shMIDN group, and IL‐1β+OE‐MIDN group, and performed quantitative ELISA assays for TNF‐α and IL‐6 (Figure 5i). The data demonstrate that MIDN is a key positive regulator of SASP secretion: IL‐1β significantly elevates extracellular levels of TNF‐α and IL‐6; MIDN knockout (shMIDN) markedly inhibits this secretion; whereas MIDN overexpression (OE‐MIDN) further enhances and accelerates the extracellular release of these inflammatory cytokines (*p* < 0.001). This directly establishes MIDN as the dominant factor underlying the paracrine pathogenic mechanism of SASP.

The rescue experiments constructed a complete chain of evidence proving that TSC2 is an indispensable downstream effector molecule in the MIDN pathogenic pathway. MIDN activation of mTORC1 is achieved not through indirect metabolic regulation but by directly reducing TSC2 protein abundance. This hierarchical cascade(MIDN↑ → TSC2↓ → mTORC1↑) establishes TSC2 as a critical checkpoint within this signaling axis, implying that maintaining TSC2 protein stability is sufficient to resist metabolic imbalance triggered by impaired CMA.

### 2.6 Impaired CMA Aggravates Rat IDD Progression

Finally, the therapeutic potential of targeting the final effector node of this signaling axis, mTORC1, was explored using the specific mTORC1 inhibitor Rapamycin (RAPA). In in vitro experiments, RAPA treatment significantly inhibited nucleus pulposus cell senescence induced by co‐treatment with IL‐1β and OE‐MIDN (Figure 6a,b) and downregulated the expression of senescence and matrix degradation markers (Figure 6c,d).

**FIGURE 6:**
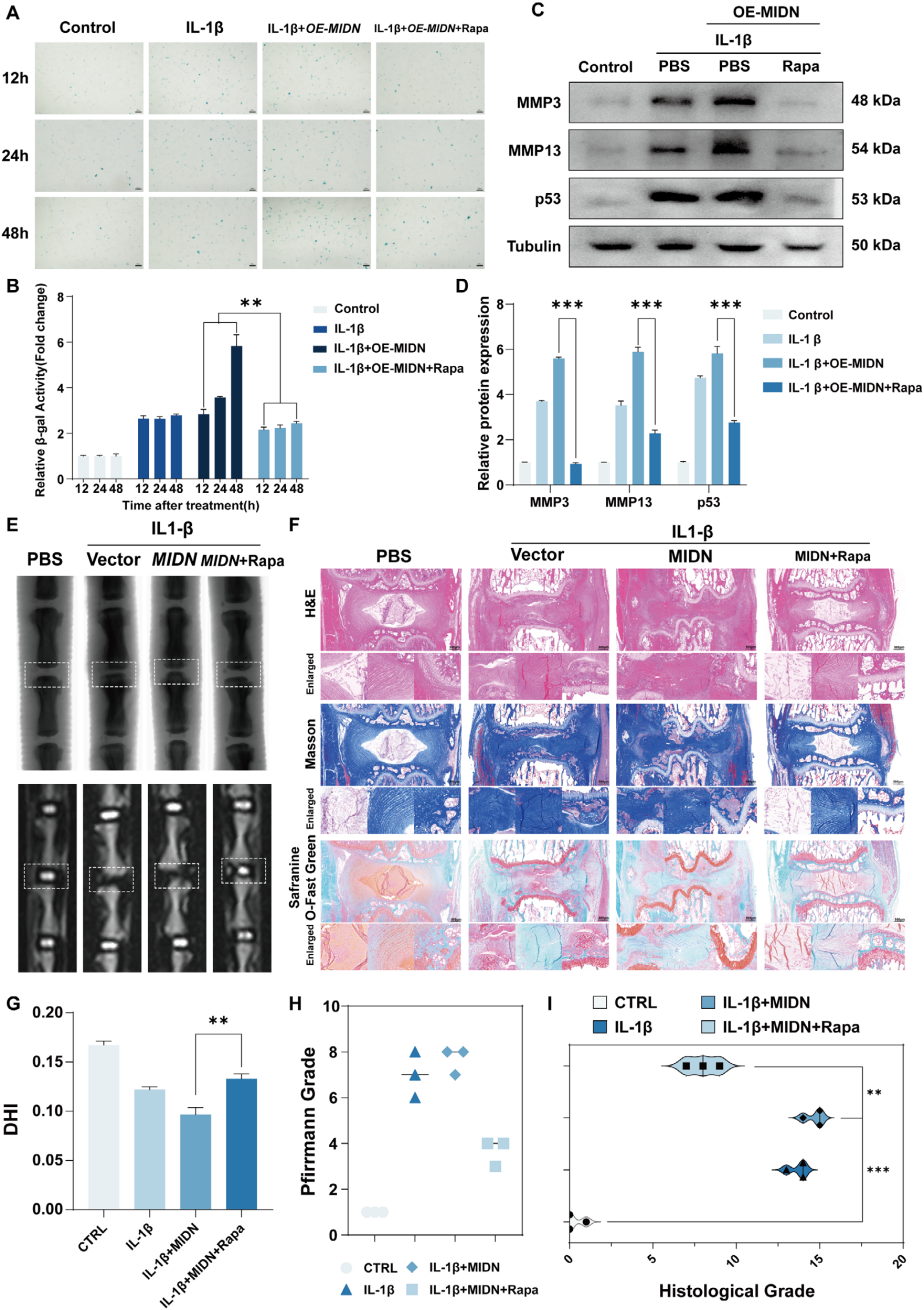
Impaired CMA Aggravates Rat IDD Progression, and mTORC1 Inhibitor Rescues this Phenotype. (a, b) SA‐β‐gal staining images (a) and fold change of positive cells (b), showing that Rapamycin alleviates cell senescence. (c, d) WB detection (c) and quantification (d) showing that Rapamycin treatment significantly inhibits the expression of MMP3, MMP13, and p53 induced by IL‐1β and OE‐MIDN. (e, g) x‐ray/MRI images (e) and DHI% quantification (g) in the rat caudal puncture model injected with PBS, Vector, OE‐MIDN, and OE‐MIDN+Rapamycin. (f) Histological staining (H&E, Masson, Safranin O‐Fast Green) of intervertebral discs in each group. (h, i) Pfirrmann grading scores based on MRI (h) and histological scores based on staining (i), showing that Rapamycin significantly improves disc degeneration caused by MIDN overexpression.

This finding was validated in the rat caudal needle puncture IDD model. Compared with the control group, intervertebral discs injected with OE‐MIDN lentivirus showed severe degeneration, including significant loss of disc height (DHI) on x‐ray and MRI (Figure 6e,g), and structural disorganization and massive proteoglycan loss shown by histological staining (Figure 6f,h,i). However, concurrent RAPA administration with OE‐MIDN effectively blocked this degenerative process, significantly maintaining disc height and ECM content, with markedly improved histological scores (Figure 6e–i).

This result provides important translational medical evidence. Since the pathological consequences of impaired CMA ultimately converge on hyperactivation of mTORC1, direct inhibition of mTORC1 can functionally “bypass” the complex upstream protein degradation defects. The significant efficacy of rapamycin in vivo indicates that for degenerated discs with already impaired CMA function, intervention at the downstream metabolic node via pharmacological means can effectively delay the degeneration process without the need for complex genetic repair of lysosomal function. This offers a highly promising intervention strategy for the clinical treatment of IDD.

## 3 Discussion

This study is the first to reveal a key downstream pathway of chaperone‐mediated autophagy (CMA) dysfunction in the pathogenesis of IDD. The research elucidates that under inflammatory (IL‐1β) stimulation, CMA activity in nucleus pulposus cells is impaired, causing its novel substrate MIDN to accumulate abnormally due to ineffective clearance. Accumulated MIDN subsequently mediates TSC2 degradation, relieving inhibition on mTORC1, and ultimately activating the mTORC1 pathway to drive cell senescence and matrix degradation, thereby aggravating IDD.

As one of the core mechanisms maintaining cellular proteostasis, CMA dysfunction is closely associated with various age‐related diseases [24, 25]. Previous studies have suggested a protective role for CMA in IDD [15]. Our study further confirms that expression of CMA key components Hsc70 and LAMP2A is downregulated in IDD patient samples and IL‐1β‐treated cells (Figure 3g), indicating that impaired CMA is a significant event in IDD pathology. Activation of CMA (e.g., LAMP2A overexpression) effectively alleviates IDD phenotypes (Figure 3j–n), suggesting CMA may serve as a potential therapeutic target for IDD [12].

One of the most important findings of this study is the identification of MIDN as a novel CMA substrate. A critical phenotype‐genotype separation phenomenon was observed in IDD: MIDN protein levels increased significantly with IDD severity (Figure 2a,b), while its mRNA levels decreased (Figure 2e). This “inversion” between transcription and post‐translation strongly points to obstacles in protein degradation pathways. Through a series of rigorous experiments, including inhibitor screening, KFERQ motif identification (DRLRQ and VERLQ) (Figure 3e), and key site mutation (Q382A/Q396A) (Figure 3o–r), we definitively confirmed that MIDN is a substrate of CMA.

The MIDN‐TSC2‐mTORC1 axis revealed in this study provides new insights into IDD pathogenesis. mTORC1 is a central hub regulating cell growth, metabolism, and senescence. Hyperactivation of mTORC1 has been confirmed as a common feature of IDD and various aging‐related diseases [9]. We found that MIDN directly targets TSC2 via its Ubl domain (Figure 4f), mediating its degradation. This process bypasses the ubiquitin‐dependent degradation pathway via the proteasome (Figure 4i–k) [16]. Biochemically, the capacity of MIDN to usher TSC2 directly into the proteasome without requiring a canonical poly‐ubiquitin chain resides in its unique bipartite structural topology, consisting of an N‐terminal Ubiquitin‐like (Ubl) domain and a C‐terminal Catch domain [26]. Emerging structural biology evidence reveals that the Ubl domain of MIDN shares high tertiary structural homology with native ubiquitin, enabling it to bypass the classic E1‐E2‐E3 enzymatic cascade and directly dock onto the non‐ATPase regulatory subunits (such as Rpn1 or Rpn2) of the 19S proteasome regulatory particle [27, 28]. Concurrently, the highly flexible C‐terminal Catch domain serves as a specialized substrate‐capture module that recognizes and anchors the solvent‐accessible peptide segments of TSC2. By acting as a non‐canonical molecular scaffold, MIDN bridges TSC2 directly to the proteasomal machinery, decreasing the entropic barrier for clearance and channeling the non‐ubiquitinated substrate straight into the 20S catalytic core for degradation. This structural cooperation provides a robust theoretical foundation for the unique proteolytic clearance pathway characterized in IDD pathology.

Importantly, the therapeutic value of targeting the mTORC1 pathway in disc degeneration has been highlighted in previous studies. For instance, Kakiuchi et al. demonstrated that local administration of Rapamycin, a classic mTORC1 inhibitor, effectively suppressed senescence markers, preserved extracellular matrix synthesis, and attenuated disc degeneration in vivo [11]. While their work established a solid proof‐of‐concept for direct mTORC1 pharmacological inhibition in IDD, the precise endogenous mechanisms driving pathological mTORC1 hyperactivation during disc degeneration had remained largely elusive. Our findings complement and expand upon this paradigm by identifying the upstream biochemical cascade responsible for this aberrant signal. We reveal that the progressive loss of CMA activity under inflammatory stress leads to the abnormal accumulation of MIDN, which continuously targets TSC2—the critical endogenous brake of mTORC1—for non‐canonical proteasomal degradation. Consequently, while direct downstream inhibition via Rapamycin offers a robust rescue effect as shown by Kakiuchi et al., our study introduces an alternative, upstream therapeutic approach. Restoring endogenous CMA activity or targeting the MIDN‐TSC2 interface may offer a more precise and homeostatic intervention, potentially mitigating the broad‐spectrum side effects associated with prolonged, direct mTORC1 inhibition.

Furthermore, MIDN accumulation was observed in both the nucleus and cytoplasm (Figure 3a,b). This study focused on its function in mediating TSC2 degradation in the cytoplasm. However, does MIDN accumulation in the nucleus have other functions (e.g., acting as a transcription factor or regulating epigenetics)? What is the mechanism of its nucleocytoplasmic shuttling? These are directions worthy of future exploration. To extend this narrative, the robust nuclear translocation and accumulation of MIDN under inflammatory stress represents a striking phenotype that cannot be decoupled from IDD progression. Structurally, MIDN contains distinct zinc‐finger‐like consensus motifs and nuclear localization signals, suggesting a potent capacity to interface with genomic or transcriptional machinery. In the context of chronic disc degeneration, nuclear‐localized MIDN may function as a transcriptional co‐activator or chromatin scaffold. It could directly bind to promoter regions of genes encoding catabolic metalloproteinases (e.g., MMP3/13) or interact with epigenetic modifiers, such as histone deacetylases (HDACs), to repress anti‐senescence loci. Thus, MIDN may act as a dual‐action pathogenic engine: it clears the post‐translational brake on mTORC1 in the cytoplasm, while simultaneously orchestrating the transcriptional amplification of downstream SASP factors within the nucleus.

Several intrinsic limitations of this study should be noted. First, our mechanistic evaluations and therapeutic interventions were primarily conducted utilizing primary rat NP cells and a small‐animal caudal puncture model. Because quadrupeds exhibit vastly different mechanical loading vectors across the spinal axis compared with bipedal humans, caution must be exercised when extrapolating these animal data to human clinical therapeutics. Future validation in large‐animal models (such as porcine or ovine disc injury models) is highly warranted. Second, while we successfully confirmed that MIDN silencing or macroautophagy modulation mitigates senescence in vitro, the long‐term biological consequences of permanent, systemic CMA impairment on adjacent vertebral endplates and annulus fibrosus tissue integrity remain to be fully elucidated.

## 4 Materials and Methods

### 4.1 Human Nucleus Pulposus Tissue Specimens

This study was approved by the Ethics Committee of Union Hospital, Tongji Medical College, Huazhong University of Science and Technology (No. UHCT‐IEC‐SOP‐016‐03‐01). All patients signed informed consent forms. Degenerated nucleus pulposus (NP) tissue specimens were collected from patients undergoing discectomy for lumbar disc herniation at the Department of Orthopedics of our hospital (*n* = 3). The degree of degeneration was assessed by preoperative T2‐weighted MRI according to the Pfirrmann grading system [29]. Non‐degenerated NP tissues (Pfirrmann Grade I‐II) were obtained from patients undergoing surgery for traumatic spinal fractures (*n* = 3).

### 4.2 Animal Models and In Vivo Treatment

Eight‐week‐old male SD rats (*n* = 3 rats per group) were purchased from Hunan Slyk Jingda Laboratory Animal Co., Ltd. All animal procedures were approved by the Institutional Animal Care and Use Committee (IACUC) of Tongji Medical College, Huazhong University of Science and Technology (No. 4904, 11/1/2025). Rats were anesthetized by intraperitoneal injection of pentobarbital sodium (50 mg/kg).

Under C‐arm x‐ray guidance, an annular puncture was performed on the target caudal discs (Co7/8 and Co8/9) using a 27G hollow needle. The needle was inserted perpendicular to the tail to a precise depth of 4 mm, held in place for 10 s, rotated 180°, and then withdrawn without aspiration. Each target disc was punctured exactly once. To establish the IDD model, IL‐1β solution was subsequently injected into the punctured discs. Notably, a puncture‐only group (subjected to the identical needle insertion protocol followed by PBS vehicle injection) was established as a critical control; this group confirmed that the mechanical puncture procedure itself did not induce disc degeneration under our experimental conditions.

One week after modeling, 2 µL of PBS containing the corresponding lentivirus (OE‐MIDN, shMIDN, OE‐LAMP2A, or control Vector) or drug (Rapamycin, 10 µm, MCE) was delivered into the target discs (Co7/8 and Co8/9) using a 33G Hamilton syringe to minimize supplementary mechanical injury. The injection volume of 2 µL was determined based on our prior pilot safety studies, which verified that an intradiscal injection volume ≤ 2.5 µL did not cause physical leakage or compromise the structural integrity of the rat disc tissue.

### 4.3 Radiographic and Histological Assesnt

X‐ray and 3.0T MRI (Siemens) scans were performed on the rat tails at 4 and 8 weeks post‐operation. The Disc Height Index (DHI) was measured and calculated based on x‐ray films according to the protocol described by Masuda et al. [30]: DHI% = (Postoperative DHI/Preoperative DHI)*100%. The degree of disc degeneration on MRI coronal T2‐weighted images was evaluated and graded based on the Pfirrmann classification system [29] on a scale of I (normal) to V (severe degeneration). After 8 weeks, rats were sacrificed, and caudal vertebral specimens were harvested. The specimens were fixed in 4% paraformaldehyde for 48 h, decalcified in 10% EDTA solution, embedded in paraffin, and cut into 5 µm thick sagittal sections. Hematoxylin‐eosin (H&E), Masson's trichrome, and Safranin O‐Fast Green (SOFG) staining were subsequently performed to evaluate structural changes. To quantitatively evaluate the severity of disc degeneration, histological slides were assessed blindly by two independent observers utilizing the ORS spine section initiative histopathological scoring system [31]. This multi‐parametric grading scheme evaluates five core categories: nucleus pulposus (NP) morphology, NP cellularity, NP‐annulus fibrosus (AF) border, AF morphology, and endplate. Each parameter was scored from 0 (completely normal/healthy) to 2 (severe degeneration), yielding a cumulative degenerative score of 0 to 14.

### 4.4 Cell Culture and Reagent Treatment

Primary nucleus pulposus cells (NPCs) were isolated from the caudal intervertebral discs of 8‐week‐old SD rats. Briefly, NP tissue was collected and digested with 0.25% trypsin (Gibco) for 30 min, followed by digestion with 0.2% Collagenase Type II (Sigma–Aldrich, #C0130) for 4 h. Cells were resuspended in DMEM/F12 medium (Gibco, #C11330500CP) containing 15% fetal bovine serum (FBS, Gibco, #10099141) and 1% penicillin/streptomycin (Gibco, #15140122) and cultured in an incubator at 37°C with 5% CO_2_.

Reagent treatment: NPCs were treated with recombinant rat IL‐1β (R&D Systems, #501‐RL‐010, 0–20 ng/mL) at various concentrations for 24 or 48 h. Other reagents included: Rapamycin (RAPA, 100 nm, MCE, #HY‐10219), Bafilomycin A1 (BafA1, 100 nm, MCE, #HY‐10058), MG132 (10 µm, MCE, #HY‐13259), Cycloheximide (CHX, 20 µg/mL, MCE, #HY‐12320), CMA activator QX77 (10 µm, MCE, #HY‐112108), and CMA inhibitor PPD (10 µm, MCE, #HY‐100118). For the CCK‐8 cell viability assay, NPCs were seeded in 96‐well plates and incubated with CCK‐8 reagent (Dojindo, Japan) for 2 h at 37°C after 24, 48, or 72 h treatments. Absorbance was read at 450 nm using a microplate reader.

### 4.5 Plasmid Construction and Lentiviral Infection

Full‐length cDNA sequences of rat Midnolin (MIDN), TSC2, and LAMP2A were cloned into the CMV‐3xFLAG‐pLVX vector (Clontech) to construct overexpression (OE) plasmids30. MIDN point mutation plasmids (Q382A, Q396A) were constructed using the KOD‐Plus‐Mutagenesis Kit (Toyobo, #SMK‐101). shRNA targeting MIDN (shMIDN) and shRNA targeting TSC2 (shTSC2) sequences were cloned into lentiCRISPRv2 (Addgene, #52961) and pLKO.1 (Addgene, #8453) vectors, respectively. The above plasmids were co‐transfected with packaging plasmids psPAX2 (Addgene, #12260) and pMD2.G (Addgene, #12259) into HEK‐293T cells (ATCC, #CRL‐3216). Viral supernatants were collected after 48 h. For infection, NPCs were incubated with viral supernatants mixed with Polybrene (8 µg/mL, Sigma, #H9268) for 24 h.

### 4.6 RNA Extraction and Real‐Time Quantitative PCR (RT‐qPCR)

Total cellular RNA was extracted using TRIzol reagent (Invitrogen, #15596026). 1 µg of RNA was reverse transcribed into cDNA using the PrimeScript RT Reagent Kit (Takara, #RR047A). RT‐qPCR was performed on a QuantStudio 7 Flex system (Applied Biosystems) using SYBR Green qPCR Master Mix (Takara, #RR820A). Relative expression levels were calculated using the 2^ΔΔCt method with GAPDH as the internal control.

**Primer sequences(5’‐3’)**:

*MIDN*
F: TACACCCCAACTGCCAAGAC
R: GGGTTGACTTCTGGCTTCCA
*TSC2*
F: TCACAGTCCTTTGAGCGGTC
R: GAGGCCATATTTGCGTGCAG
*P53*
F: CTGGCCCCTGTCATCTTCTG
R: ATTCTCTTCCTCTGTGCGCC
*MMP3*
F: AGTCTTCCAATCCTACTGTTGCT
R: TCCCCGTCACCTCCAATCC
*MMP13*
F: ATGCAGCAAGCTCCATGACT
R: GGGTCCTTGGAGTGGTCAAG
*COL2A1*
F: CTTCTGGCCCTAGAGGTCCT
R: GCTGCTCCACCAGTTCTTCT
*ACAN*
F: CAGTGAGACGTCCGCCTATC
R: GGTACTTGTTCCAGCCCTCC
*TNFα*
F: TCAGCCTCTTCTCCTTCCTGA
R: CGAGATAGTCGGGCCGATTG
*IL‐6*
F: CACAGACAGCCACTCACCTC
R: ATTTGCCGAAGAGCCCTCAG
*IL‐8*
F: ATGACTTCCAAGCTGGCCG
R: GGGTCCAGACAGAGCTCTCT
*ACTIN*
F: CACCATTGGCAATGAGCGGTTC
R: AGGTCTTTGCGGATGTCCACGT
*mTOR*
F: GAACGCTTGGCAGCTTTGAA
R: CATGTAGGGGCGGATGAGTC
*LAMP2A*
F:AACTTCCTTGTGCCCATAGC
R:AGCATGATGGTGCTTGAGAC
*Hsc70*
F:AGAACAAGAGAGCTGTCCGC
R:TCCTTGGTCATGGCCCTTTC

### 4.7 Enzyme‐Linked Immunosorbent Assay (ELISA)

To quantify the extracellular secretion levels of key senescence‐associated secretory phenotype (SASP) factors, cell culture supernatants from primary rat nucleus pulposus cells (NPCs) in different treatment groups (Control, IL‐1β [20 ng/mL], IL‐1β + shMIDN, and IL‐1β + OE‐MIDN) were collected after 48 h of incubation. The supernatants were centrifuged at 2,000 × g for 10 min at 4°C to remove residual cellular debris and stored at −80°C until further analysis. Extracellular secretion concentrations of tumor necrosis factor‐alpha (TNF‐α) and interleukin‐6 (IL‐6) were quantitatively measured using commercial Rat TNF‐α ELISA Kits and Rat IL‐6 ELISA Kits (Abcam, Cambridge, UK) in accordance with the manufacturer's instructions. Optical densities (OD values) were measured at 450 nm using a microplate reader (Bio‐Rad Laboratories, Hercules, CA, USA). All assays were performed with three independent biological replicates, and the secretion concentrations were calculated against standard curves.

### 4.8 RNA Sequencing (RNA‐Seq) and Bioinformatics Analysis

Total RNA was extracted from the OE‐MIDN group and control group (*n* = 3/group) using the RNeasy Mini Kit (Qiagen, #74104). Sequencing libraries were constructed using the NEBNext Ultra RNA Library Prep Kit (NEB, #E7530L) and sequenced on the Illumina NovaSeq 6000 platform. Differentially expressed genes (DEGs) analysis was performed using the DESeq2 R package (padj < 0.05, |Log2(Fold Change)| > 1). GO functional enrichment, KEGG pathway analysis, and Gene Set Enrichment Analysis (GSEA) were conducted using the ClusterProfiler R package.

### 4.9 Proteomics and Mass Spectrometry (MS) Analysis

1. Total Proteomics (TMT): Total protein was extracted from the OE‐MIDN and control groups (*n* = 3/group). After trypsin digestion, peptides were labeled using the TMTpro 16plex Label Reagent Set (Thermo Fisher, #A44520). Peptides were analyzed by LC‐MS/MS (Q Exactive HF, Thermo Fisher Scientific). Proteins were identified and quantified by searching the UniProt rat database using MaxQuant software (v1.6.17.0). Differentially expressed proteins (DEPs) were screened and subjected to GO/KEGG enrichment analysis. A Protein‐Protein Interaction (PPI) network was constructed using the STRING database (v11.5) and Cytoscape software (v3.8.2).
2. Immunoprecipitation‐Mass Spectrometry (IP‐MS): NPCs lysates were immunoprecipitated using Anti‐FLAG M2 Affinity Gel (Sigma, #A2220) or anti‐MIDN antibody (Abcam). Eluates were subjected to short SDS‐PAGE electrophoresis, Coomassie Blue staining, gel excision, and enzyme digestion, followed by LC‐MS/MS analysis to identify interacting proteins.

### 4.10 Western Blot and Nuclear‐Cytoplasmic Fractionation

Cells were lysed in RIPA lysis buffer (Beyotime, #P0013B), and protein concentration was quantified using a BCA kit (Thermo Fisher, #23227). Protein samples were separated by SDS‐PAGE and transferred to PVDF membranes (Millipore, #ISEQ00010). Membranes were blocked in 5% non‐fat milk for 1 h and incubated with primary antibodies overnight at 4°C. After incubation with HRP‐conjugated secondary antibodies (CST) for 1 h, bands were visualized using ECL chemiluminescent substrate (Millipore, #WBKLS0500). Nuclear‐cytoplasmic fractionation was performed using the NE‐PER Nuclear and Cytoplasmic Extraction Reagents (Thermo Fisher, #78833).

AntibodiesThe primary antibodies used in this study were as follows: anti‐Midnolin (MIDN) [Zenbio, #860621], anti‐Hsc70 [Zenbio, #R24618], and anti‐LAMP2A [Zenbio, #R24835] for autophagy and CMA analysis; anti‐TSC2 [Proteintech, #24601‐1‐AP], anti‐mTOR [Proteintech, #66888‐1‐Ig], anti‐p70 S6 Kinase (S6K) [Proteintech, #14485‐1‐AP], anti‐phospho‐p70 S6 Kinase (Thr389) [Proteintech, #82373‐1‐RR], anti‐S6 Ribosomal Protein [Proteintech, #80208‐1‐RR], anti‐phospho‐S6 Ribosomal Protein (Ser235/236) [Proteintech, #29223‐1‐AP], anti‐4E‐BP1 [Proteintech, #60246‐1‐Ig], and anti‐phospho‐4E‐BP1 (Thr37/46) [Proteintech, #81812‐4‐RR] for mTORC1 pathway detection. For the assessment of senescence and inflammation, anti‐p53 [Zenbio, #345567] and anti‐TNF‐α [Zenbio, #346654] were used. ECM‐related markers were detected using anti‐MMP3 [Zenbio, #340612], anti‐MMP13 [Zenbio, #R381531], and anti‐Collagen II (COL2A1) [Zenbio, #222189]. Loading controls and tags included anti‐GAPDH [Proteintech, #60004‐1‐Ig], anti‐β‐Tubulin [Proteintech, #10094‐1‐AP], anti‐Histone H2 [Proteintech, #39209], and anti‐FLAG [Zenbio, #T201126‐3A6]. For immunoprecipitation controls, Normal Rabbit IgG [Zenbio, #550060] was used. Secondary antibodies conjugated with HRP for Western blot were obtained from [Zenbio, #550017, #550060], and Alexa Fluor 488/594‐conjugated secondary antibodies for immunofluorescence were purchased from [ThermoFisher, #A‐11008, #A‐11001, #A‐21207, #A‐11005].

### 4.11 Immunofluorescence (IF) and Confocal Microscopy

Cell slides were fixed with 4% paraformaldehyde (PFA) for 15 min, permeabilized with 0.1% Triton X‐100 for 10 min, and blocked with 5% BSA for 1 h. Slides were then incubated with primary antibodies (MIDN, TSC2, Hsc70) overnight at 4°C. The next day, slides were incubated with Alexa Fluor 488‐ or 594‐labeled secondary antibodies for 1 h at room temperature, and nuclei were counterstained with DAPI. Images were acquired using a Leica SP8 laser scanning confocal microscope. Colocalization analysis was performed using ImageJ to calculate the Pearson correlation coefficient (R value).

### 4.12 Co‐Immunoprecipitation (Co‐IP)

Cells were lysed in IP lysis buffer (Thermo Fisher, #87787)43. 500 µg of total protein was incubated with 2 µg of anti‐MIDN, anti‐Hsc70, anti‐TSC2, or rabbit IgG antibody overnight at 4°C with rotation. Protein A/G Plus‐Agarose beads (Thermo Fisher, #26147) were then added and incubated for another 4 h. After thorough washing, beads were boiled in loading buffer and subjected to Western Blot detection.

### 4.13 Cell Senescence and Cell Cycle Analysis

44SA‐β‐gal staining: Cell senescence was detected using the Senescence β‐Galactosidase Staining Kit (Cell Signaling Technology, #9860)45. Cell cycle analysis: Cells were collected and fixed with 75% ice‐cold ethanol overnight at 4°C. Cells were stained with PI/Triton X‐100 staining solution containing RNase A (BD Biosciences, #550825) for 30 min at room temperature in the dark. Cell cycle distribution was analyzed using a FACS Canto II flow cytometer (BD Biosciences).

### 4.14 Statistical Analysis

All in vitro experiments were performed with at least three independent biological replicates. Data analysis was conducted using GraphPad Prism 9.0 software (GraphPad Software, Inc.), and results are presented as Mean ± Standard Deviation (SD). Prior to statistical comparisons, the normality and homoscedasticity of the datasets were systematically evaluated using the Shapiro‐Wilk test and Brown‐Forsythe test, respectively. For normally distributed data, statistical significance between two groups was determined using a two‐tailed unpaired Student's t‐test. For multiple group comparisons, a one‐way or two‐way analysis of variance (ANOVA) was performed, followed by Tukey's or Sidak's post hoc multiple comparison tests. A two‐tailed p‐value < 0.05 was considered statistically significant.

## 5 Conclusions

In summary, this study systematically identified and elucidated a novel signaling pathway playing a key driving role in intervertebral disc degeneration (IDD). The study reveals that under inflammatory stress (e.g., IL‐1β) environments, chaperone‐mediated autophagy (CMA) function in nucleus pulposus cells (NPCs) is impaired, leading to the abnormal cytoplasmic accumulation of its novel substrate Midnolin (MIDN) due to ineffective clearance. Crucially, this accumulated MIDN gains a “toxic function,” directly binding to and mediating the degradation of TSC2, the core inhibitor of the mTORC1 pathway, via its Catch/Ubl domains.

Notably, this degradation process bypasses the classic ubiquitin‐proteasome system and relies instead on the proteasomal pathway. This not only provides a new perspective for TSC2 regulation but also reveals a new mode of non‐ubiquitin‐dependent protein degradation. The depletion of TSC2 ultimately leads to excessive activation of the mTORC1 signaling pathway, driving senescence, SASP phenotypes, and extracellular matrix (ECM) degradation in NPCs, collectively accelerating the pathological progression of IDD.

The significance of this study lies in its first‐time direct linkage of a specific defect in proteostasis (impaired CMA) with a key regulatory hub of cell metabolism and senescence (mTORC1) in the context of IDD. This provides a novel upstream mechanism explaining why mTORC1 is abnormally activated in IDD.

More importantly, this “CMA–MIDN–TSC2–mTORC1” signaling axis offers multiple highly potential therapeutic targets for IDD intervention. Both in vitro and in vivo experiments confirmed that activating CMA (e.g., LAMP2A overexpression), knocking down MIDN (shMIDN), or using the mTORC1 inhibitor Rapamycin all effectively alleviated intervertebral disc degeneration phenotypes induced by the abnormal activation of this pathway. In particular, the significant protective effect of Rapamycin in animal models further highlights the clinical translational value of targeting this pathway, providing a solid theoretical foundation and a promising new direction for developing etiological treatment strategies for IDD (such as developing specific CMA agonists or MIDN inhibitors).

## Author Contributions

**Xianglong Chen**: conceptualization, methodology, validation, writing – original draft, project administration, visualization. **Wang Wu**: writing – review and editing, resources, software. **Wenbo Wu**: validation, visualization, project administration. **Pengzhi Shi**: methodology, supervision, project administration. **Zhangrong Cheng**: conceptualization, methodology, validation, project administration, writing – review and editing. **Yuhang Chen**: investigation, validation, visualization. **Zimu Yu**: formal analysis, writing – review and editing, data curation. **Yukun Zhang**: supervision, resources, funding acquisition, project administration. **Anran Zhang**: conceptualization, project administration. **Haiyang Gao**: conceptualization, methodology, data curation, supervision, writing – review and editing.

## Funding

This study was supported by grants from the National Natural Science Foundation of China (No. 82172497).

## Conflicts of Interest

The authors declare no conflicts of interest.

## Supporting information




**Supporting File**: advs77428‐sup‐0001‐SuppMat.docx.

## Data Availability

The data that support the findings of this study are available from the corresponding author upon reasonable request.
