# Supplementary material for: Impaired Chaperone‐Mediated Autophagy Accelerates Intervertebral Disc Degeneration by Inducing MIDN Accumulation to Target TSC2 for Proteasomal Degradation
Source: Adv Sci (Weinh). 2026 Aug 27:e77428. Online ahead of print. doi: 10.1002/advs.77428 (PMC13521188; doi:10.1002/advs.77428)
Supplement: Supplementary file 1 [file ADVS-9999-e77428-s001.docx]

****Figure S1. MIDN regulates inflammatory markers, cellular senescence, and intervertebral disc degeneration in vitro and in vivo (Supplemental).****

**(a) RT-qPCR analysis of relative mRNA expression levels of MIDN, p53, MMP3, MMP13, COL2A1, ACAN, TNF-α, IL-6, and IL-8 in rat NPCs under WT, IL-1β (20 ng/mL) stimulation, IL-1β + OE-MIDN, or IL-1β + shMIDN conditions. (b) Relative β-galactosidase activity (fold change) at 12 h, 24 h, and 48 h in NPCs under Ctrl, EV, OE-MIDN, or shMIDN transfection with IL-1β treatment. (c) Flow cytometry histograms showing cell cycle distribution and phase percentages of NPCs in each group. (d) Pfirrmann grade of caudal discs in the CTRL, Puncture, IL-1β, IL-1β + OE-MIDN, and IL-1β + shMIDN groups at 8 weeks post-operation in rats. (e) Histological grade of discs in each experimental group at 8 weeks post-operation based on ORS score. Data represent Mean ± SD, n=3. *P < 0.05, **P < 0.01, ***P < 0.001.**

****Figure S2. Interaction and spatial colocalization between MIDN and TSC2 (Supplemental).****

**(a) Pearson's correlation coefficient (R value) quantifying the spatial colocalization between MIDN and TSC2 in NPCs under Control (Ctrl) and IL-1β stimulation conditions. (b) Schematic model illustrating the non-canonical, ubiquitin-independent proteasomal degradation of TSC2 mediated by Midnolin (MIDN). Under inflammatory stress, CMA dysfunction leads to the abnormal accumulation of MIDN in the cytoplasm. MIDN subsequently binds directly to TSC2 via its Catch domain and delivers it to the proteasome core via its Ubl domain, triggering mTORC1 hyperactivation, NPC senescence, and disc degeneration. Data represent Mean ± SD, n=3. **P < 0.01.**
